# Perfluoroalkyl substances in umbilical cord blood and blood pressure in offspring: a prospective cohort study

**DOI:** 10.1186/s12940-023-01023-5

**Published:** 2023-10-19

**Authors:** Zhikang Xu, Bowen Du, Hualin Wang, Zhuoyan Li, Yujian Wu, Qianchuo Wang, Yiwei Niu, Qianlong Zhang, Kun Sun, Jian Wang, Sun Chen

**Affiliations:** 1 Department of Pediatric Cardiology, Xinhua Hospital, Shanghai Jiao Tong University School of Medicine, No.1665, Kongjiang Road, Yangpu District, Shanghai, 200092 China; 2 Ministry of Education-Shanghai Key Laboratory of Children’s Environmental Health, Xinhua Hospital, Shanghai Jiao Tong University School of Medicine, Shanghai, China

**Keywords:** Perfluoroalkyl substances (PFAS), Intrauterine exposure, Vascular function, Blood pressure, Birth cohort, Umbilical cord blood

## Abstract

**Background:**

Humans are widely exposed to perfluoroalkyl substances (PFAS), which have been found to be associated with various adverse birth outcomes. As blood pressure (BP) is an important parameter reflecting cardiovascular health in early life, it is necessary to investigate the association of PFAS exposure during early lifetime and BP in childhood. Therefore, we investigated the potential association between PFAS levels in umbilical cord blood and BP of the offspring at 4 years of age in a prospective cohort study.

**Methods:**

PFAS in umbilical cord blood samples after birth were measured with high-performance liquid chromatography/tandem mass spectrometry in the Shanghai Birth Cohort. BP was measured at 4 years of age in the offspring. Multiple linear regression model was used to investigate the association between individual PFAS level and BP of the offspring. Bayesian kernel machine regression (BKMR) was used to analyze the relationship between the PFAS mixture and BP of the offspring, while weighted quantile sum (WQS) regression was utilized for sensitivity analysis.

**Results:**

A total of 129 mother-child pairs were included in our analysis. In multiple linear regressions, we observed that long-chain PFAS, mainly including perfluorooctane sulfonate (PFOS), perfluorodecanoic acid (PFDA) and perfluoroundecanoic acid (PFUA), was negatively associated with systolic blood pressure (SBP), diastolic blood pressure (DBP) and mean arterial blood pressure (MAP). BKMR showed that an increase in umbilical cord blood PFAS mixture levels was significantly associated with a decrease in SBP, DBP and MAP [Estimated differences (SD): -0.433 (0.161); -0.437 (0.176); -0.382 (0.179), respectively]. The most important component in the association with SBP, DBP, and MAP was PFUA. PFDoA was found to be positively associated with SBP, DBP and MAP in both models. Sensitivity analysis with WQS regression showed consistent results.

**Conclusion:**

Our findings suggested that umbilical blood PFAS exposure was negatively associated with BP in offspring at 4 years of age, including SBP, DBP, and MAP.

**Supplementary Information:**

The online version contains supplementary material available at 10.1186/s12940-023-01023-5.

## Background

Perfluoroalkyl substances (PFAS) are a series of organic fluoride compounds, which contain one or more carbon with fluorine in place of hydrogen atoms [1]. Because of their chemical and thermal stability, they are widely used in industrial processes and the production of consumer goods. Humans can be exposed through the air, food, and water [2]. Meanwhile, these physicochemical properties resulted in a long half-life and bioaccumulation in human bodies and the environment [3]. PFAS could cross the placenta and be detected in umbilical cord blood, which might harm fetal development [4].

According to the developmental origins of health and disease (DOHaD) hypothesis, the abnormal intrauterine environment and prenatal exposure can lead to abnormal offspring development and an increased risk of diseases during adulthood [5]. As a widely existing organic compound, PFAS exposure has been found to be associated with a lot of adverse birth outcomes, including low birth weight, preterm birth, intrauterine growth restriction [3, 6], thyroid dysfunction [7], and nervous dysplasia [8]. As blood pressure (BP) is an important parameter of early life cardiovascular health, it is important to investigate the association between PFAS exposure and BP. However, in terms of the correlation between PFAS exposure and children’s BP, current evidences remain controversial. Two studies have found serum PFAS levels in children were positively associated with their BP [9, 10], while four other studies have showed that neither prenatal nor postnatal PFAS exposure was associated with BP of the offspring [11–14]. Furthermore, the concentrations of PFAS in umbilical cord blood reflects the exposure level of the fetus after filtration through the placental barrier [15–17], which might provide additional evidence for early life time PFAS exposure, and there has been no evidence about the association between PFAS levels in umbilical cord blood and offspring BP. Therefore, since these inconsistent and relative deficient evidences, further investigations between umbilical cord blood PFAS exposure and offspring BP levels are in need.

This study aimed to explore the association between individual and mixture PFAS exposure in umbilical cord blood with offspring’s BP in early childhood.

## Materials and methods

### Study design and participants

The present analysis was based on the Shanghai Birth Cohort (SBC). A detailed description of the cohort was previously published [18]. In brief, 255 mother-child pairs with data of PFAS levels in umbilical cord blood were recruited between 2013 and 2016 at 6 SBC participating hospitals [19]. At admission to the study, structured questionnaires were filled in with participants’ basic characteristics, medical records, lifestyle with the assist of well-trained staff. Regular follow-ups were carried out for anthropometric indicators of children including weight, height, and body mass index (BMI) from birth to 4 years of age. The 4-year -old follow-up was conducted from 2018 to 2021. Cord blood samples were collected after birth. Among the 255 mother-child pairs, after exclusion of participants with loss-to-follow-up, miscarriages, stillbirths, and lack of BP data at 4 years old, 129 offspring accomplished BP measurements at 4 years old. This research was approved by the Research Ethics Committees from Xinhua Hospital affiliated to Shanghai Jiao Tong University School of Medicine (XHEC-C-3-001-3). Written informed consents were acquired from all parents or guardians of participants before enrollment.

### PFAS Concentration Measurement

Cord blood samples were collected after birth and immediately centrifuged and frozen at − 80 °C. A detailed description of the collective and analytical method was published elsewhere [20]. In brief, a total of 10 targeted PFAS, including perfluorooctanate (PFOA), perfluorodecanoic acid (PFDA), perfluorooctane sulfonate (PFOS), perfluorohexanesulfonate (PFHxS), perfluorononanoic acid (PFNA), perfluoroundecanoic acid (PFUA), perfluorobutane sulfonate (PFBS), perfluoroheptanoic acid (PFHpA), perfluorododecanoic acid (PFDoA) and perfluorooctane sulfonamide (PFOSA) were quantified in 100 µl plasma using high-performance liquid chromatography/tandem mass spectrometry (HPLC/MS-MS; Agilent1290–6490, Agilent Technologies Inc., USA). PFOSA was not included in the further analysis as it was detected in only 52.7% of samples while other PFAS substances were detected in all samples. The intra-assay coefficients of variation (CV) were between 0.8 and 8.5% and the inter-assay CV were between 1.7 and 8.4% [21].

### BP measurements

Systolic BP (SBP) and diastolic BP (DBP) of the children were assessed in the supine position by trained staff members on the left arm at heart level with the appropriate cuff size for arm circumference by the OMRAN HBP-1300 automatic BP device (Omron Healthcare, Guangzhou, China) [22, 23]. After the child relaxed, three measurements were taken at 5-min intervals. The mean of the last 2 measurements was used in all analyses. The mean arterial BP (MAP) was calculated by the formula [MAP=(SBP + DBP×2)/3]. Pulse pressure (PP) was calculated as SBP - DBP.

### Statistics analysis

In order to increase the normality of the data, ln-transformation of PFAS concentrations was conducted. The assessment of the bivariate correlation among the different PFAS substances was carried out by Spearman correlation and cluster analysis (Figure S1).

The assessment of the relationship between individual PFAS chemical and BP was applied by a multiple linear regression model. Model 1 was adjusted for household income, educational levels, hypertensive disorder complicating pregnancy (HDP), gestational diabetes mellitus (GDM), drinking history, passive smoking history, and maternal age. Model 2 was adjusted for birthweight, sex, BMI at 4 year of age, and confounder adjusted in Model 1 [24–27].

The assessment of the combined effects of the PFAS mixture and BP was conducted by the nonparametric Bayesian kernel machine regression (BKMR) with the R packages of “bkmr”. The BKMR flexibly models the exposure-response relationship with a Gaussian kernel function [28]. The differences between all PFAS levels fixed at a specific quartile compared to their 50th percentile after a total of 20,000 iterations indicated the combined effects of PFAS mixture in umbilical cord blood on BP, which was presented as estimated differences and standard deviation (SD). The weights of the effect on the outcome were represented by estimated conditional posterior inclusion probabilities (condPIPs) of different PFAS substances. The model was adjusted for household income, educational levels, GDM, HDP, drinking history, passive smoking history, maternal age, birthweight, sex and BMI of children.

Weighted quantile sum (WQS) regression was conducted using the ‘gWQS’ packages [29]. A total of 10,000 bootstrap samples were generated from the full data set and used to estimate weights for each PFAS. The WQS index was used to estimate the combined effect of the PFAS mixture on BP. The corresponding average weight of each PFAS was calculated to identify the important component.

Nonlinear association was investigated with restricted cubic spline (RCS) based on four knots of PFAS levels using ‘ggrcs’ R packages. RCS models were adjusted for household income, educational levels, GDM, HDP, drinking history, passive smoking history, maternal age, birthweight, sex and BMI of children. Tests for non-linearity were conducted by using analysis of variance tests. Sex-subgroup analysis was performed with multiple linear regression models and adjusted for the same covariates. An interactive effect analysis of individual PFAS level and sex was also conducted in subgroup analysis.

All the analyses were performed using the STATA software, version 15.0 (Stata Corporation, College Station, TX, USA) and R version 4.0.4 (R Foundation for Statistical Computing) with p < 0.05 were considered statistically significant.

## Results

The general characteristics of the study population are presented in Table 1. The mean (SD) maternal age was 31.2 (3.1) years old. Most mothers had a bachelor’s degree and nearly half had a household income ≥ 100,000 RMB/year. There are 5 (3.9%) mothers who had HDP and 12 (9.3%) mothers who had GDM during pregnancy. The mean gestational age, birth weight, birth height and BMI of the offspring at 4 years old was 38.9 (1.3) weeks, 3.4 (0.4) kg, 50.0 (1.0) cm and 15.0 (1.5) kg/m^2^, respectively. The mean SBP, DBP, MAP, and PP were 98.4 (7.9), 57.5 (6.2), 71.2 (5.8), and 41.2 (6.3), respectively.

**Table 1:** Baseline characteristics of participants

Variables	Overall (N = 129)
**Mother characteristics**	
Maternal age (years), Mean ± SD	31.2 ± 3.1
Household income (RMB/year), N(%)	
≥100,000	65(50.4)
< 100,000	64(49.6)
Educational level, N(%)	
≥ Bachelors’ degree	107(82.9)
< Bachelors’ degree	22(17.1)
GDM, N(%)	12(9.3)
HDP, N(%)	5(3.9)
Drinking during pregnancy, N(%)	21(16.3)
Passive smoking during pregnancy, N(%)	34(26.4)
**Offspring characteristics**	
Weight at 4y (kg), Mean ± SD	17.9 ± 2.8
Height at 4y (cm), Mean ± SD	108.8 ± 4.8
BMI at 4y (kg/m^2^), Mean ± SD	15.0 ± 1.5
Gestational age (weeks), Mean ± SD	38.9 ± 1.3
Birth weight (kg), Mean ± SD	3.4 ± 0.4
Birth height (cm), Mean ± SD	50.0 ± 1.0
Sex, N(%)	
Boy	69(53.5)
Girl	60(46.5)
Blood pressure at 4y (mmHg), Mean ± SD	
SBP	98.4 ± 7.9
DBP	57.5 ± 6.2
MAP	71.2 ± 5.8
PP	41.2 ± 6.3

BMI: body mass index, SBP: systolic blood pressure, DBP: diastolic blood pressure, MAP: mean artery pressure, PP: pulse pressure, HDP: hypertensive disorders in pregnancy, GDM: gestational diabetes mellitus

The distribution of PFAS detected in umbilical cord blood was described with median and inter-quartile range. The limit of detection (LOD) and the detected rate of each PFAS were shown in Table 2. The highest median concentration of PFAS in umbilical blood was PFOA (7.29ng/ml), followed by PFOS (3.14ng/ml), PFNA (0.63ng/ml), PFHxS(0.46ng/ml), PFDA (0.39ng/ml), PFUA (0.37ng/ml), PFOSA (0.17ng/ml), PFBS (0.13ng/ml), PFHpA (0.12ng/ml), PFDoA (0.09ng/ml). Nine PFAS had detection rates of 100%, except for PFOSA, which was excluded from the further analyses. The PFAS concentrations were highly correlated (Figure S1).

**Table 2:** Distribution of PFAS concentrations in umbilical cord blood plasma (ng/ml) (N = 129)

	LOD	>LOD%	25th percentile	Median	75th percentile	Minimum	Maximum	Mean	SD
PFOA	0.09	100	5.59	7.29	10.84	2.09	24.61	8.59	4.47
PFOS	0.09	100	2.15	3.14	4.39	0.66	22.43	4.00	3.25
PFNA	0.02	100	0.44	0.63	0.81	0.24	2.48	0.70	0.38
PFDA	0.02	100	0.29	0.39	0.57	0.14	2.85	0.50	0.37
PFUA	0.02	100	0.27	0.37	0.55	0.12	2.20	0.46	0.30
PFHxS	0.02	100	0.31	0.46	0.88	0.10	3.23	0.66	0.53
PFHpA	0.03	100	0.09	0.12	0.16	0.05	0.29	0.14	0.05
PFOSA	0.12	52.7	0.01	0.17	0.18	0.01	0.34	0.10	0.09
PFDoA	0.05	100	0.06	0.09	0.13	0.02	0.42	0.10	0.06
PFBS	0.01	100	0.12	0.13	0.15	0.10	0.24	0.14	0.03

PFOA: perfluorooctanate, PFOS: perfluorooctane sulfonate, PFNA: perfluorononanoic acid, PFDA: perfluorodecanoic acid, PFUA: perfluoroundecanoic acid, PFHxS: perfluorohexanesulfonate, PFHpA: perfluoroheptanoic acid, PFOSA: perfluorooctane sulfonamide, PFDoA: perfluorododecanoic acid, PFBS: perfluorobutane sulfonate

Multiple linear regression models were used to investigate the association between individual umbilical PFAS concentration and BP of the offspring. PFOS had a negative correlation with SBP after confounders adjustment (PFOS: β=-3.10, 95%CI: -5.20, -0.89). In terms of DBP, we found that PFDA, PFOS, and PFUA were inversely associated with DBP (PFDA: β=-2.31, 95%CI: -4.56, -0.06; PFOS: β=-2.15, 95%CI: -4.04, -0.33; PFUA: β=-2.48, 95%CI: -4.91, -0.11). Besides, PFDA and PFOS were also negatively correlated with MAP (PFDA: β=-2.30, 95%CI: -4.42, -0.22; PFOS: β=-1.96, 95%CI: -3.72, -0.24). Meanwhile, we also found that PFDoA and PFHpA showed a positive trend with BP of offspring (Table 3).

As there might be a sex disparity of the influence of PFAS [30], we further conducted a sex subgroup analysis and an interactive effect analysis of PFAS and sex with multiple linear regression models. It indicated that the negative association of PFDA, PFOS and PFUA with BP was mainly showed in boys, while the positive association of PFDoA and PFHpA with BP was mainly showed in girls (Table S3). Besides, we found that in terms of SBP, DBP and MAP, the interactive effect was generally not significant, only PFDoA and sex showed a significant interactive effect in the association between PFDoA and SBP. Moreover, long-chain PFAS showed a significant interactive effect with sex in the association between them and PP (Table S3).

**Table 3:** Association of PFAS concentrations in umbilical cord blood plasma with children BP in Early childhood

	SBP			DBP			MAP			PP		
	Crude model	Model 1	Model 2	Crude model	Model 1	Model 2	Crude model	Model 1	Model 2	Crude model	Model 1	Model 2
PFDA	**-2.91(-5.52,-0.28)**	**-3.30(-6.04,-0.51)**	-2.50(-5.14,0.16)	**-2.24(-4.43,0.00)**	**-2.33(-4.63,-0.06)**	**-2.31(-4.56,-0.06)**	**-2.41(-4.45,-0.40)**	**-2.63(-4.78,-0.53)**	**-2.30(-4.42,-0.22)**	-0.68(-3.04,1.68)	-0.93(-3.41,1.56)	-0.18(-2.51,2.15)
PFDoA	2.16(-0.45,4.77)	1.91(-0.84,4.65)	2.08(-0.56,4.71)	1.34(-0.87,3.56)	1.08(-1.19,3.34)	1.15(-1.11,3.41)	1.16(-0.88,3.20)	0.94(-1.18,3.07)	1.06(-1.05,3.18)	0.82(-1.52,3.15)	0.83(-1.60,3.26)	0.92(-1.38,3.22)
PFHpA	**4.15(0.43,7.83)**	**4.12(0.20,8.09)**	3.24(-0.58,7.06)	2.91(-0.23,6.05)	2.34(-0.94,5.61)	2.29(-0.98,5.55)	2.71(-0.18,5.60)	2.46(-0.60,5.53)	2.04(-1.03,5.11)	1.22(-2.12,4.55)	1.81(-1.72,5.33)	0.95(-2.39,4.30)
PFHxS	-1.18(-3.17,0.81)	-1.16(-3.21,0.89)	-1.46(-3.42,0.51)	-0.21(-1.90,1.48)	-0.44(-2.13,1.25)	-0.61(-2.30,1.07)	-0.11(-1.66,1.44)	-0.17(-1.76,1.42)	-0.42(-2.00,1.16)	-0.97(-2.73,0.79)	-0.71(-2.52,1.09)	-0.84(-2.55,0.87)
PFNA	-2.25(-5.22,0.72)	-2.41(-5.52,0.70)	-2.18(-5.11,0.76)	-1.67(-4.18,0.84)	-1.83(-4.39,0.73)	-2.00(-4.50,0.49)	-2.06(-4.35,0.23)	-2.28(-4.67,0.10)	-2.27(-4.59,0.06)	-0.58(-3.23,2.07)	-0.58(-3.34,2.18)	-0.18(-2.74,2.39)
PFOA	-1.39(-4.25,1.46)	-1.27(-4.20,1.66)	-1.63(-4.43,1.17)	-0.66(-3.08,1.76)	-0.45(-2.87,1.96)	-0.76(-3.15,1.64)	-0.79(-3.01,1.44)	-0.66(-2.93,1.61)	-1.04(-3.29,1.20)	-0.73(-3.27,1.80)	-0.82(-3.40,1.77)	-0.87(-3.30,1.56)
PFOS	**-3.69(-5.79,-1.56)**	**-3.84(-6.06,-1.61)**	**-3.10(-5.20,-0.89)**	**-2.45(-4.30,-0.67)**	**-2.27(-4.13,-0.38)**	**-2.15(-4.04,-0.33)**	**-2.33(-4.02,-0.69)**	**-2.31(-4.08,-0.58)**	**-1.96(-3.72,-0.24)**	-1.19(-3.14,0.76)	-1.58(-3.62,0.45)	-0.86(-2.79,1.07)
PFUA	**-3.05(-5.77,-0.36)**	**-3.64(-6.54,-0.78)**	-2.49(-5.33,0.34)	**-2.52(-4.83,-0.27)**	**-2.72(-5.12,-0.37)**	**-2.48(-4.91,-0.11)**	**-2.50(-4.56,-0.37)**	**-2.75(-5.02,-0.58)**	-2.16(-4.42,0.09)	-0.52(-2.96,1.93)	-0.91(-3.52,1.69)	0.02(-2.47,2.50)
PFBS	5.99(-1.53,13.51)	5.99(-1.77,13.75)	3.28(-4.28,10.84)	-0.59(-6.99,5.82)	-0.89(-7.32,5.55)	-0.89(-7.35,5.57)	2.10(-3.78,7.98)	1.94(-4.10,7.98)	0.77(-5.29,6.83)	6.58(-0.04,13.19)	**6.84(0.10,13.65)**	4.17(-2.35,10.68)

Multiple linear regression models were used and adjusted for maternal and offspring factors

Model 1: adjusted for household income, educational levels, GDM, HDP, drinking history, passive smoking history and age of mom

Model 2: Model 1 + birthweight, sex and BMI of children

PFOA: perfluorooctanate, PFOS: perfluorooctane sulfonate, PFNA: perfluorononanoic acid, PFDA: perfluorodecanoic acid, PFUA: perfluoroundecanoic acid, PFHxS: perfluorohexanesulfonate, PFHpA: perfluoroheptanoic acid, PFDoA: perfluorododecanoic acid, PFBS: perfluorobutane sulfonate; BMI: body mass index, SBP: systolic blood pressure, DBP: diastolic blood pressure, MAP: mean artery pressure, PP: pulse pressure, HDP: hypertensive disorders in pregnancy, GDM: gestational diabetes mellitus

**Fig. 1:**
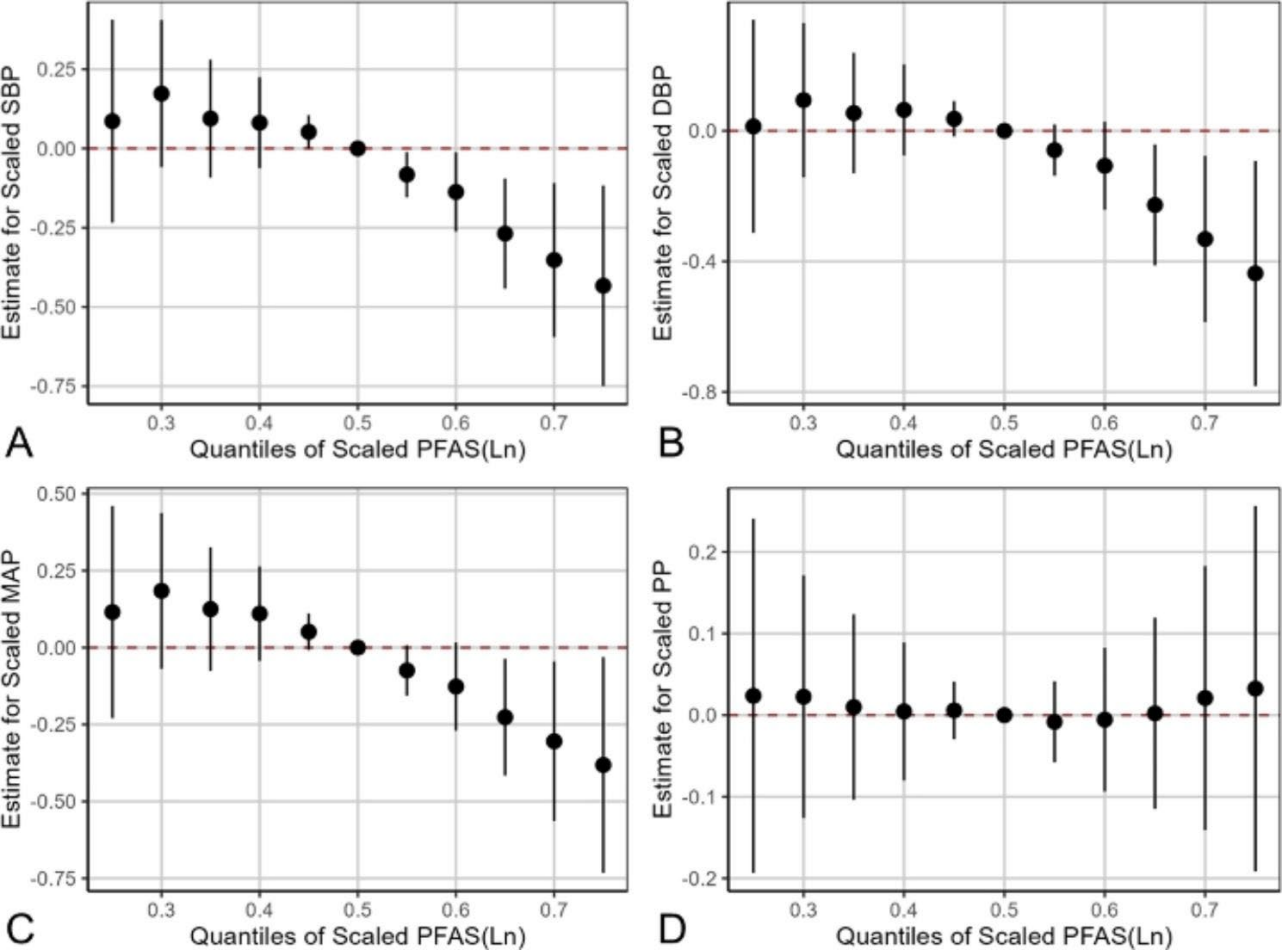
Joint associations of umbilical cord blood PFAS mixture (ln-transformed) with children BP (A. SBP, B. DBP, C. MAP, D. PP) estimated by Bayesian kernel machine regression (BKMR) (N = 129). The figure plotted the estimated differences and 95% CIs in the BP when all the PFAS were fixed at a specific quantile (from 0.25 to 0.75) compared to all the PFAS at their 50th percentile. The results were adjusted for household income, educational levels, GDM, HDP, drink history, passive smoke history, age of mom, birthweight, sex and BMI of children. SBP: systolic blood pressure, DBP: diastolic blood pressure, MAP: mean artery pressure, PP: pulse pressure, BMI: body mass index, HDP: hypertensive disorders in pregnancy, GDM: gestational diabetes mellitus

The PFAS mixture in cord blood was significantly associated with lower SBP, DBP and MAP, but not with PP. When all PFAS mixture were fixed at the 75th percentile, in comparison to at their 50th percentile, SBP, DBP and MAP significantly decreased [Estimated differences (SD): -0.433 (0.161); -0.437 (0.176); -0.382 (0.179) respectively) (Fig. 1, Table S1). To characterize the contribution of individual PFAS chemical to the overall effect, we further estimated the association of an inter-quartile range increase of each PFAS on BP when others were set at different percentile levels [29]. Generally, all the PFAS substances showed a negative correlation with SBP, DBP and PP, except that PFDoA showed a positive association with them (Figure S2). The main contribution of the effect of PFAS on SBP, DBP, and MAP was PFUA (condPIP = 0.871, condPIP = 0.753, condPIP = 0.684, respectively) (Table 4), with an inversed relationship (Figure S2). Among all the PFAS substances in umbilical blood, the effect of PFUA on SBP and DBP was significant when all of the other PFAS were fixed at 25th and 50th percentile levels, except that when all of the other PFAS were fixed at 75th, the correlation became not significant. (Figure S3). In general, the univariate exposure-response relationship analysis in BKMR showed the consistent results with multiple linear regression models.

**Table 4:** Estimated conditional posterior inclusion probabilities (condPIPs) of umbilical cord blood PFAS (ln-transformed) in relation with children BP in BKMR analyses

	SBP	DBP	MAP	PP
PFBS	0.275	0.437	0.451	0.214
PFDA	0.424	0.470	0.538	0.236
PFDoA	0.925	0.730	0.773	0.217
PFHpA	0.348	0.500	0.446	0.205
PFHxS	0.237	0.407	0.354	0.229
PFNA	0.309	0.514	0.508	0.217
PFOA	0.269	0.396	0.348	0.193
PFOS	0.341	0.516	0.456	0.186
PFUA	0.871	0.753	0.684	0.234

The results were adjusted for household income, educational levels, GDM, HDP, drink history, passive smoke history, age of mom, birthweight, sex and BMI of children. PFOA: perfluorooctanate, PFOS: perfluorooctane sulfonate, PFNA: perfluorononanoic acid, PFDA: perfluorodecanoic acid, PFUA: perfluoroundecanoic acid, PFHxS: perfluorohexanesulfonate, PFHpA: perfluoroheptanoic acid, PFDoA: perfluorododecanoic acid, PFBS: perfluorobutane sulfonate; BMI: body mass index, SBP: systolic blood pressure, DBP: diastolic blood pressure, MAP: mean artery pressure, PP: pulse pressure, HDP: hypertensive disorders in pregnancy, GDM: gestational diabetes mellitus

In WQS regression models, the WQS index was negatively associated with BP in general (Table S2), which was similar to the results of BKMR. Likewise, the directions of the contribution of PFUA were consistent with BKMR (Figure S4).

In terms of nonlinearity, most PFAS substances didn’t show a nonlinear association with children’s BP. Only PFBS showed an inverse U-shape curve with DBP (P for nonlinearity = 0.008) and MAP (P for nonlinearity = 0.017) (Figure S5-S8).

## Discussion

This study aimed to investigate the individual and combined effects of multiple PFAS in umbilical cord blood and BP in offspring. In individual models, PFOS showed a negative correlation with SBP. PFDA, PFOS and PFUA were inversely associated with DBP. PFDA and PFOS were also negatively correlated with MAP. In mixture analysis, a significantly inverse association of PFAS mixture in umbilical cord blood with SBP, DBP and MAP of offspring was found, using both BKMR and WQS regression. PFDoA showed a positive association with SBP, DBP and MAP in both multiple linear regression models and BKMR.

To our knowledge, this was the first prospective cohort study that reported the negative association between multiple PFAS in umbilical cord blood and BP in offspring. Currently, there have been a series of studies on the impact of individual PFAS exposure on gestational age, fetal development [31], neurodevelopment [32], behavioral problem [33], endocrine function [34, 35], and immune function [36]. But the relationship between PFAS and BP is inconsistent. Two prospective cohort studies have found no significant association between individual PFAS exposure and BP of offspring [11, 12]. Furthermore, cross-sectional studies also had also found no association between children’s serum PFAS concentration and their own BP level [13, 14]. However, two studies had found that children’s serum PFAS concentration was positively correlated with elevated BP [9, 10]. For adults, one study has found a significantly positive association between early pregnancy plasma PFAS levels and gestational hypertension [37], while another study has found a positive but not significant trend [38]. In the pre-diabetic population, a small but significant positive association of PFOA and N-methyl-perfluorooctane sulfonamido acetic acid (MeFOSAA) with BP has been found [39].

Furthermore, most current studies focused on individual PFAS substances and BP of children, mainly focused on long-chain PFAS including PFOS, PFOA, PFNA, PFDA and PFUA [9–14], which lack studies on mixture PFAS exposure during pregnancy. Our study revealed that PFAS mixture exposure in umbilical cord blood was negatively associated with SBP, DBP and MAP in 4-year-old young children, while the most important contributor to the combined effect was PFUA. As far as we know, there has been no evidence on the association between PFUA and BP of the offspring. Besides, our study also found the sex disparity of the effect of umbilical PFAS levels and offspring BP, which has not been explored in previous studies [9, 10]. Negative association between PFDA, PFOS and PFUA with BP was mainly showed in boys, while the positive association of PFDoA and PFHpA with BP was mainly showed in girls.

PFAS, as a kind of organic chemical pollutant with diverse and complicated structures, have different permeability and toxicity for humans [40–43]. The length of the carbon chain of PFAS can strongly affect their toxicity. As such, long-chain PFAS is more toxic than short-chain PFAS [41]. Our results demonstrated that the inverse association between PFAS and BP of the offspring mainly resulted from long-chain PFAS. Long-chain PFAS exposure, especially PFDA, PFOS, and PFUA were significantly negatively associated with BP of the offspring, among which PFUA contributed mainly to the impact of the mixed PFAS exposure. However, the short-chain PFAS showed no significant association with BP of the offspring. Moreover, we also found that there was a positive trend between PFDoA and BP of the offspring with both multiple linear regression and BKMR. Only one cohort study has investigated the association between individual PFDoA levels in maternal serum and the risk of gestational hypertension, and demonstrated an inverse relationship, which is inconsistent with our results [44]. Thus, further studies with larger sample size, multiple regions and detailed evaluation of BP are in need.

As BP is affected by cardiac output, cardiac function, and peripheral vascular resistance. SBP, DBP, and MAP are influenced by cardiac output and function, while PP reflects the stiffness of the peripheral blood vessels. Our previous study found that prenatal PFAS exposure might be associated with a decrease in left ventricular wall thickness, intraventricular septum thickness [45]. Therefore, we speculated that PFAS might influence BP by reducing cardiac output or function but not by vascular resistance [46]. To further investigate the potential mechanism behind the negative association between PFAS exposure and BP in offspring, a group of animal experiments were conducted. One study using zebrafish as a study model has shown that PFOA exposure during the embryonic development might result in low heart rate and affect the contraction of the myocardial cell (MC) by increasing the apoptosis of MC [47]. Another study using mouse model to investigate PFOA exposure during pregnancy has shown that PFOA exposure may cause microcardia probably through mitochondrial dysfunction induced by reactive oxygen species (ROS) release [48]. Low heart rate, reduction of the cardiac chambers, and impairment of the contraction of MC might affect the contraction of the heart and lead to reduced BP as follow. Moreover, as one kind of endocrine disruptor chemical (EDCs), PFAScna affect BP through disrupted endocrine function. A cross-species with molecular dynamics has suggested that the binding affinity of PFOS and estrogen receptor-α(ER-α) is much stronger in human beings, which signified that PFAS might combine with ER-α [49]. A series of studies have demonstrated that PFAS could mimic the function of estrogen and activate the estrogen-responsive gene expression [50, 51], while estrogen could serve as a vasodilator and lower BP [52].

Our study was the first to evaluate the association between PFAS concentrations in umbilical cord blood and blood pressure in the offspring. Most of researches focused on the hypertension but studies with results of low blood pressure rarely reported. That might be the reason that exploration on PFAS and blood pressure was so few. Besides, we used multiple PFAS levels as exposure and evaluated both mixed exposure and individual exposure. We chose PFAS levels in umbilical cord blood, which eliminated the impact of the placental barrier and were representative of the real exposed situation *in utero.*

Nevertheless, we still had several limitations in our study. First, we only investigated the relationship between umbilical cord blood PFAS levels and the BP in offspring. As a prospective cohort study conducted in Shanghai, China, the volunteers were mainly focused on the long-term residents in Shanghai, which might lead to a selection bias of the regions, occupations, and nations. Besides, the lack of postnatal PFAS exposure of the offspring was a potential study limitation. One study found that postnatal PFAS exposure through lactation accounted for a proportion of the total perinatal PFAS exposure, which revealed that postnatal PFAS exposure should be taken into concern [53]. Further research, with detailed pediatric echocardiography, a larger sample, and multiple regions included, would be helpful to validate our findings.

## Conclusions

Our prospective cohort study demonstrated that in individual analysis, long-chain PFAS showed an inverse association with BP of the offspring, which was mainly found in boys, while PFDoA showed a positive correlation, while was mainly found in girls. In mixture analysis, higher umbilical cord PFAS levels were associated with decreased BP of the offspring. Similarly, long-chain PFAS, especially PFUA, played a dominant role in this negative effect. These findings may have essential public health implications for the management of BP of 4-year-old children. Therefore, it might be important to inspect PFAS exposure during early life time, which has an association with cardiovascular health later in life.

### Electronic supplementary material

Below is the link to the electronic supplementary material.

Supplementary Material 1

## Data Availability

Deidentified individual participant data (including data dictionaries) will be made available, in addition to study protocols, the statistical analysis plan, and the informed consent form. The data will be made available upon publication to researchers who provide a methodologically sound proposal for use in achieving the goals of the approved proposal. The datasets used and/or analysed during the current study are available from the corresponding author on reasonable request. Proposals should be submitted to wangjian@xinhuamed.com.cn.

## Abbreviations

PFAS: Perfluoroalkyl substances
DOHaD: developmental origins of health and disease
BP: blood pressure
SBC: Shanghai Birth Cohort
BMI: body mass index
PFOA: perfluorooctanate
PFOS: perfluorooctane sulfonate
PFNA: perfluorononanoic acid
PFDA: perfluorodecanoic acid
PFUA: perfluoroundecanoic acid
PFHxS: perfluorohexanesulfonate
PFOSA: perfluorooctane sulfonamide
PFBS: perfluorobutane sulfonate
PFDoA: perfluorododecanoic acid
PFHpA: perfluoroheptanoic acid
CV: coefficients of variation
SBP: systolic blood pressure
DBP: diastolic blood pressure
MAP: mean arterial pressure
PP: pulse pressure
GDM: gestational diabetes mellitus
HDP: hypertensive disorder complicating pregnancy
BKMR: Bayesian kernel machine regression
CI: confidential intervals
condPIPs: conditional posterior inclusion probabilities
WQS: weighted quantile sum
GH: gestational hypertension
MeFOSAA: N-methyl-perfluorooctane sulfonamido acetic acid
MC: myocardial cell
ROS: reactive oxygen species
EDCs: endocrine disruptor chemical
ER-α: estrogen receptor-α
