# Supplementary material for: Perfluoroalkyl substances in umbilical cord blood and blood pressure in offspring: a prospective cohort study
Source: Environ Health. 2023 Oct 19;22:72. doi: 10.1186/s12940-023-01023-5 (PMC10585876; doi:10.1186/s12940-023-01023-5)
Supplement: Supplementary file 1 — Supplementary Material 1 [file 12940_2023_1023_MOESM1_ESM.docx]

**Supplementary materials**


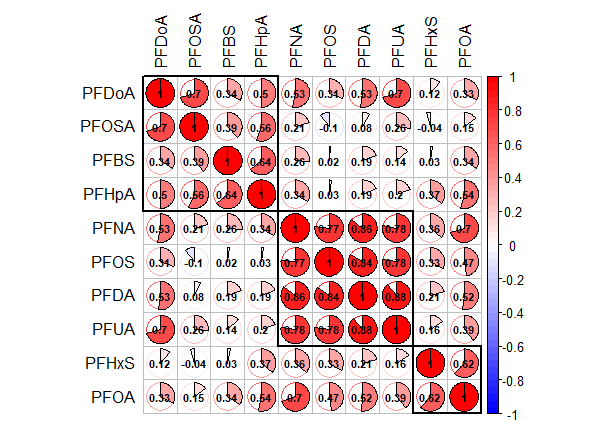


**Figure S1.** The Spearman correlations between PFAS congeners (ln-transformed).

The size and color of circles and the number represents the Spearman coefficients. H-cluster analysis was used to analyse the group the PFAS mixture. PFNA, PFOS, PFDA, PFUA were highly correlated with each other.

PFOA: perfluorooctanate, PFOS: perfluorooctane sulfonate, PFNA: perfluorononanoic acid, PFDA: perfluorodecanoic acid, PFUA: perfluoroundecanoic acid, PFHxS: perfluorohexanesulfonate, PFHpA: perfluoroheptanoic acid, PFOSA: perfluorooctane sulfonamide, PFDoA: perfluorododecanoic acid, PFBS: perfluorobutane sulfonate.


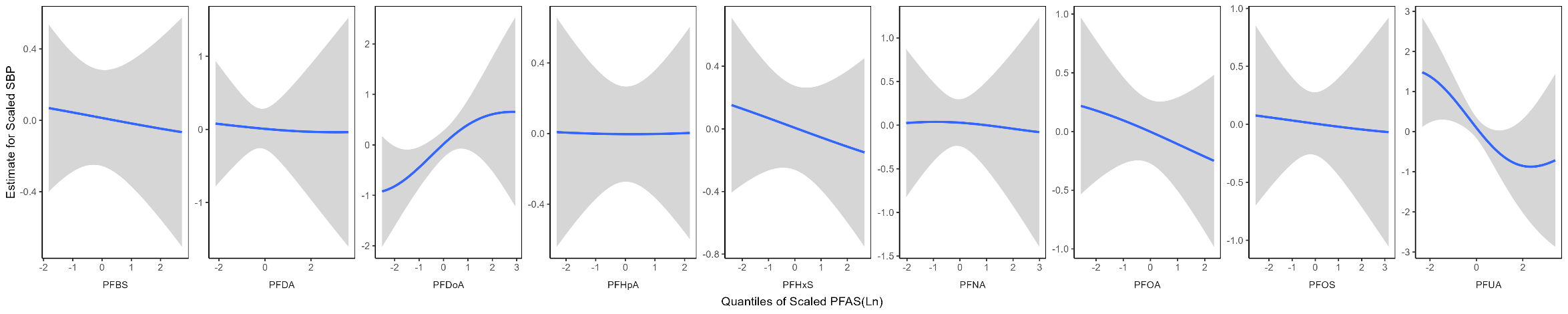


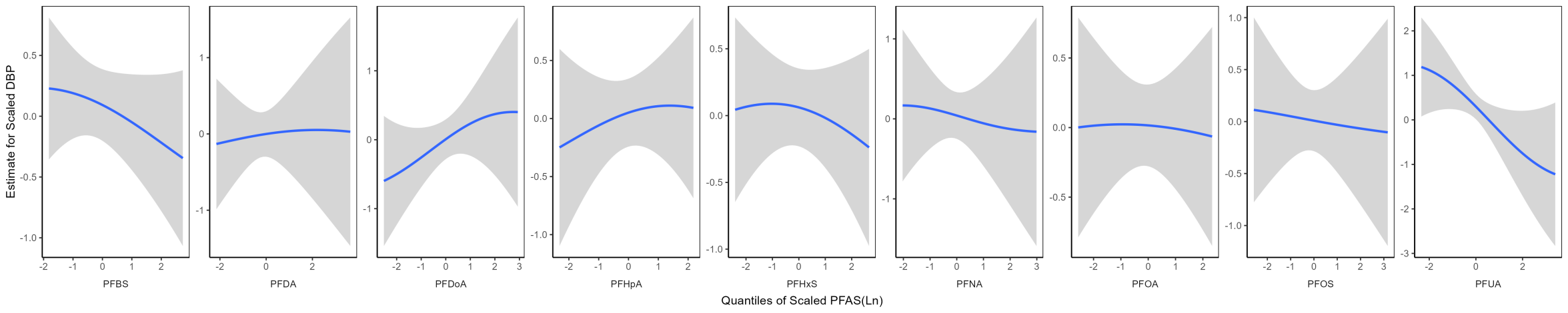

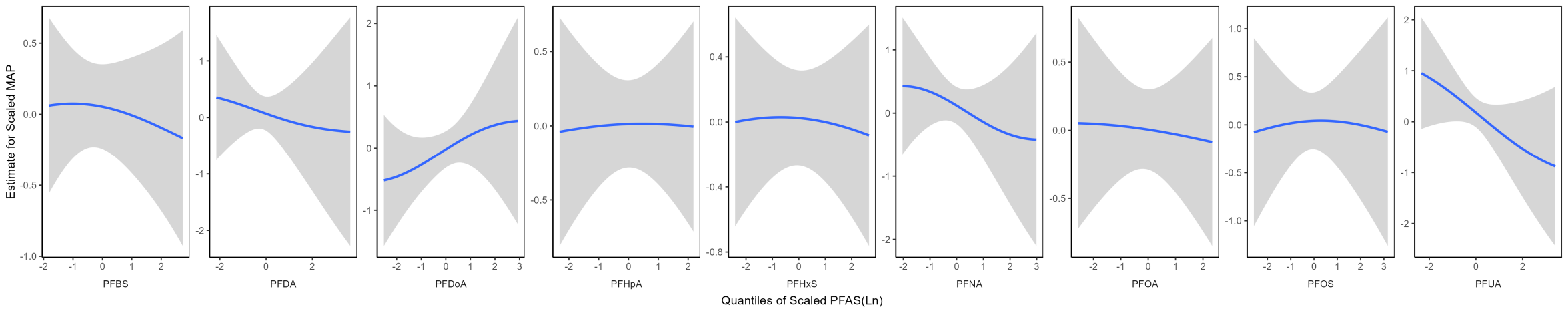

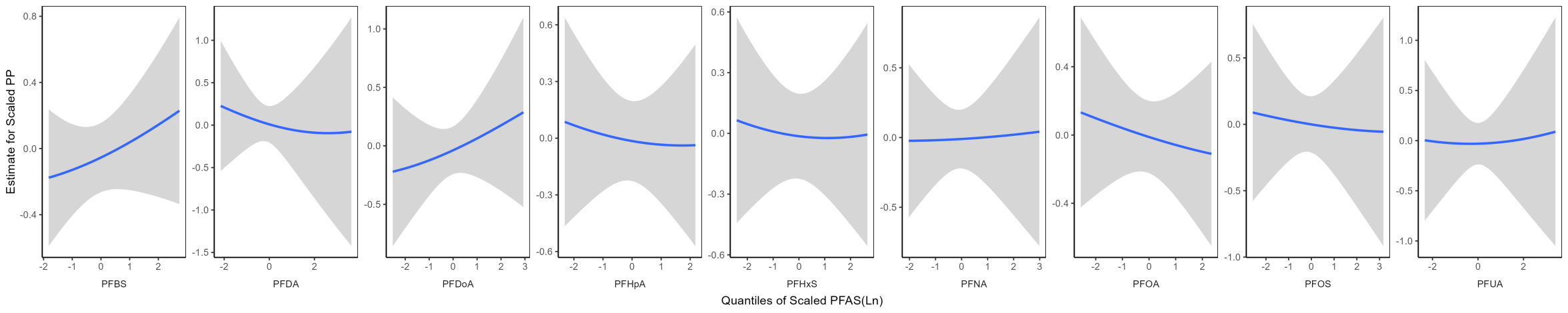


**Figure S2.** Univariate exposure-response (ER) relationships of umbilical cord blood individual PFAS (ln-transformed) chemicals with BP of children in BKMR.

The results were adjusted for household income, educational levels, GDM, HDP, drink history, passive smoke history, age of mom, birthweight, sex and BMI of children. PFOA: perfluorooctanate, PFOS: perfluorooctane sulfonate, PFNA: perfluorononanoic acid, PFDA: perfluorodecanoic acid, PFUA: perfluoroundecanoic acid, PFHxS: perfluorohexanesulfonate, PFHpA: perfluoroheptanoic acid, PFDoA: perfluorododecanoic acid , PFBS: perfluorobutane sulfonate, BMI: body mass index, SBP: systolic blood pressure, DBP: diastolic blood pressure, MAP: mean artery pressure, PP: pulse pressure, HDP: hypertensive disorders in pregnancy, GDM: gestational diabetes mellitus.

**Table S1.** The estimate overall risk of umbilical cord blood PFAS mixture in different quantile and BP in BKMR models.

|  | SBP | | DBP | | MAP | | PP | |
| --- | --- | --- | --- | --- | --- | --- | --- | --- |
| Quantile | Estimate | SD | Estimate | SD | Estimate | SD | Estimate | SD |
| 0.25 | 0.086 | 0.163 | 0.013 | 0.167 | 0.115 | 0.176 | 0.194 | 0.676 |
| 0.30 | 0.173 | 0.118 | 0.094 | 0.121 | 0.184 | 0.129 | 0.185 | 0.470 |
| 0.35 | 0.094 | 0.095 | 0.054 | 0.094 | 0.125 | 0.102 | 0.100 | 0.354 |
| 0.40 | 0.081 | 0.073 | 0.064 | 0.071 | 0.110 | 0.079 | 0.059 | 0.260 |
| 0.45 | 0.052 | 0.027 | 0.037 | 0.028 | 0.051 | 0.030 | 0.042 | 0.109 |
| 0.50 | 0.000 | 0.000 | 0.000 | 0.000 | 0.000 | 0.000 | 0.000 | 0.000 |
| 0.55 | -0.083 | 0.037 | -0.059 | 0.040 | -0.074 | 0.042 | -0.066 | 0.158 |
| 0.60 | -0.137 | 0.064 | -0.107 | 0.069 | -0.127 | 0.073 | -0.055 | 0.277 |
| 0.65 | -0.268 | 0.089 | -0.227 | 0.094 | -0.226 | 0.097 | -0.016 | 0.373 |
| 0.70 | -0.352 | 0.124 | -0.332 | 0.130 | -0.305 | 0.132 | 0.080 | 0.510 |
| 0.75 | -0.433 | 0.161 | -0.437 | 0.176 | -0.382 | 0.179 | 0.158 | 0.713 |

The estimated differences and standard deviation (SD) in the cardiovascular parameters when all the PFAS were fixed at a specific quantile (from 0.25 to 0.75) compared to all the PFAS at their 50th percentile. The results were adjusted for household income, educational levels, GDM, HDP, drink history, passive smoke history, age of mom, birthweight, sex and BMI of children. SBP: systolic blood pressure, DBP: diastolic blood pressure, MAP: mean artery pressure, PP: pulse pressure, BMI: body mass index, HDP: hypertensive disorders in pregnancy, GDM: gestational diabetes mellitus.


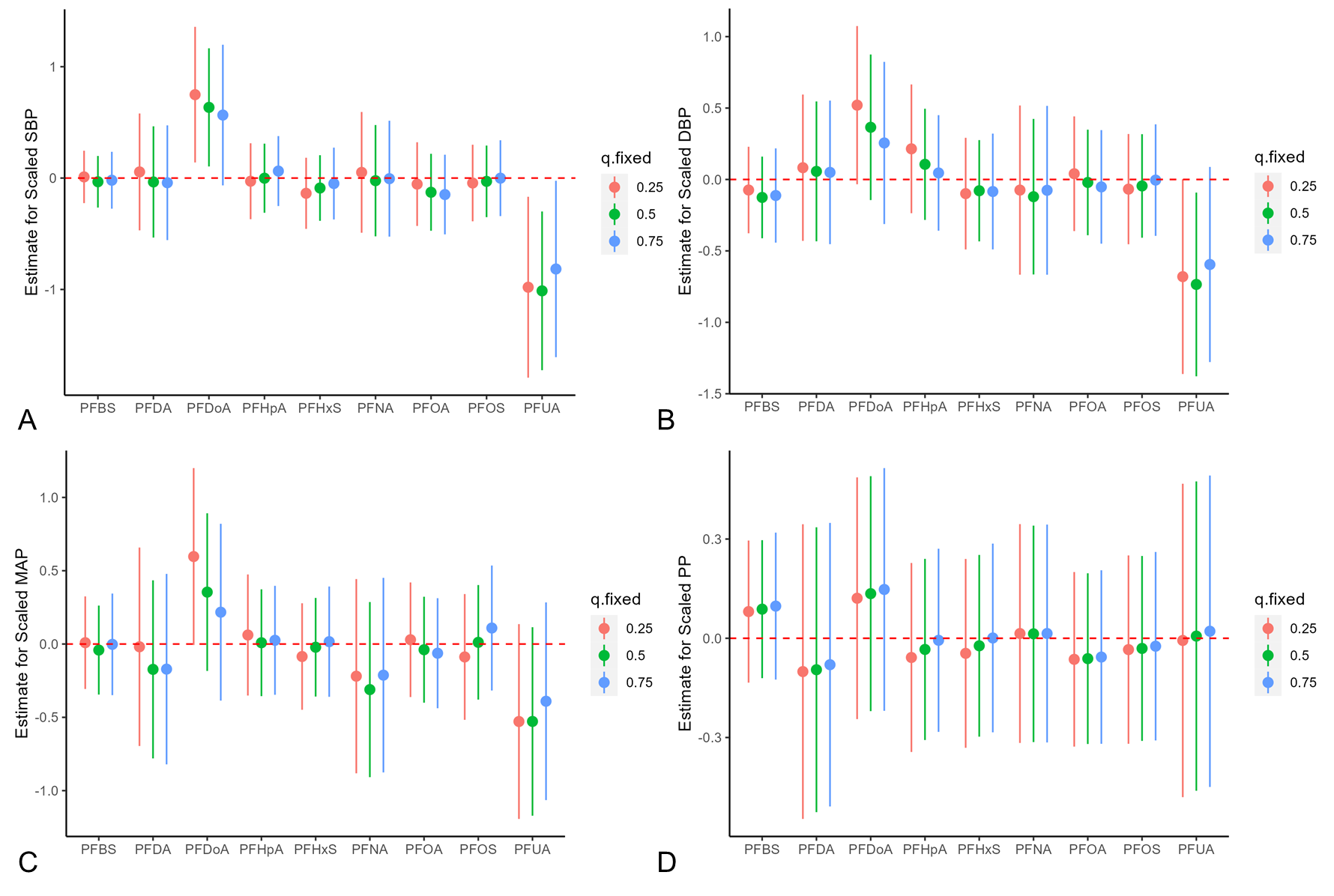


**Figure S3.** Single exposure effects of umbilical cord blood individual PFAS chemicals with BP (A. SBP, B. DBP, C. MAP, D. PP) of children in BKMR.

The results were adjusted for household income, educational levels, GDM, HDP, drink history, passive smoke history, age of mom, birthweight, sex and BMI of children. For example, that when exposure PFDoA increases from its 25th to its 75th percentile (and the other exposures remain fixed at their 75th percentile), the estimate of scaled MAP decreases by 0.4 units. PFOA: perfluorooctanate, PFOS: perfluorooctane sulfonate, PFNA: perfluorononanoic acid, PFDA: perfluorodecanoic acid, PFUA: perfluoroundecanoic acid, PFHxS: perfluorohexanesulfonate, PFHpA: perfluoroheptanoic acid, PFDoA: perfluorododecanoic acid, PFBS: perfluorobutane sulfonate, BMI: body mass index, SBP: systolic blood pressure, DBP: diastolic blood pressure, MAP: mean artery pressure, PP: pulse pressure, HDP: hypertensive disorders in pregnancy, GDM: gestational diabetes mellitus.


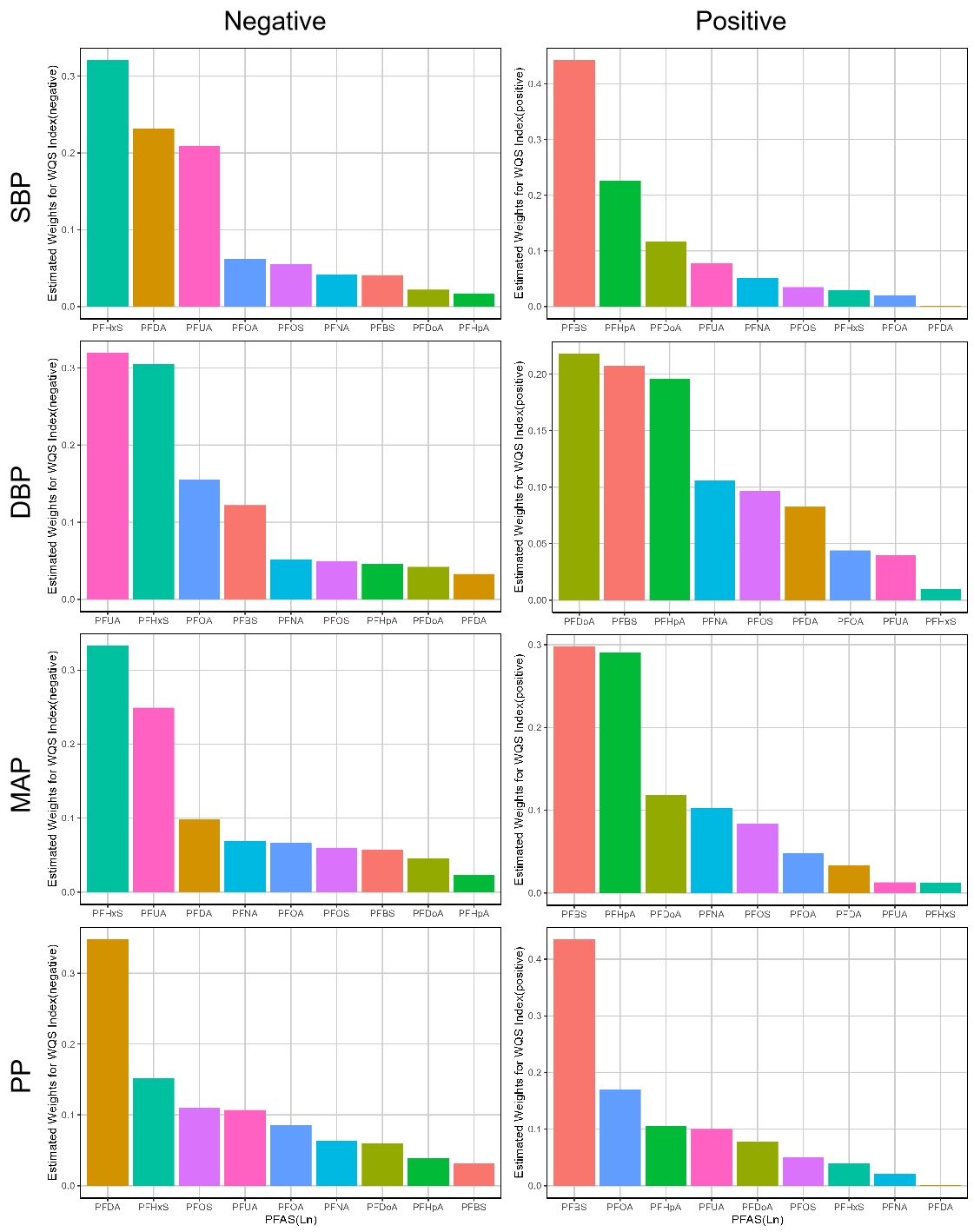


**Figure S4.** Weighted quantile sum (WQS) regression index weights of PFAS for children BP estimated in the study population.

WQS regression models were constrained the association between PFAS exposure in umbilical cord blood and BP in the negative direction. WQS models were adjusted for household income, educational levels, GDM, HDP, drink history, passive smoke history, age of mom, birthweight, sex and BMI of children.

PFOA: perfluorooctanate, PFOS: perfluorooctane sulfonate, PFNA: perfluorononanoic acid, PFDA: perfluorodecanoic acid, PFUA: perfluoroundecanoic acid, PFHxS: perfluorohexanesulfonate, PFHpA: perfluoroheptanoic acid, PFDoA: perfluorododecanoic acid, PFBS: perfluorobutane sulfonate, BMI: body mass index, SBP: systolic blood pressure, DBP: diastolic blood pressure, MAP: mean artery pressure, PP: pulse pressure, HDP: hypertensive disorders in pregnancy, GDM: gestational diabetes mellitus.

**Table S2.** The negative and positive WQS index of umbilical PFAS and children BP.

|  | **Negative WQS(SD)** | **P value** | **Positive WQS(SD)** | **P value** |
| --- | --- | --- | --- | --- |
| **SBP** | -1.470(0.967) | 0.037 | 0.228(0.935) | 0.808 |
| **DBP** | -1.200(0.91) | 0.193 | -0.969(0.881) | 0.276 |
| **MAP** | -1.140(0.793) | 0.156 | -0.576(0.771) | 0.458 |
| **PP** | 0.711(0.829) | 0.395 | 0.771(0.821) | 0.352 |

WQS models were adjusted for household income, educational levels, GDM, HDP, drink history, passive smoke history, age of mom, birthweight, sex and BMI of children. BMI: body mass index, SBP: systolic blood pressure, DBP: diastolic blood pressure, MAP: mean artery pressure, PP: pulse pressure, HDP: hypertensive disorders in pregnancy, GDM: gestational diabetes mellitus.


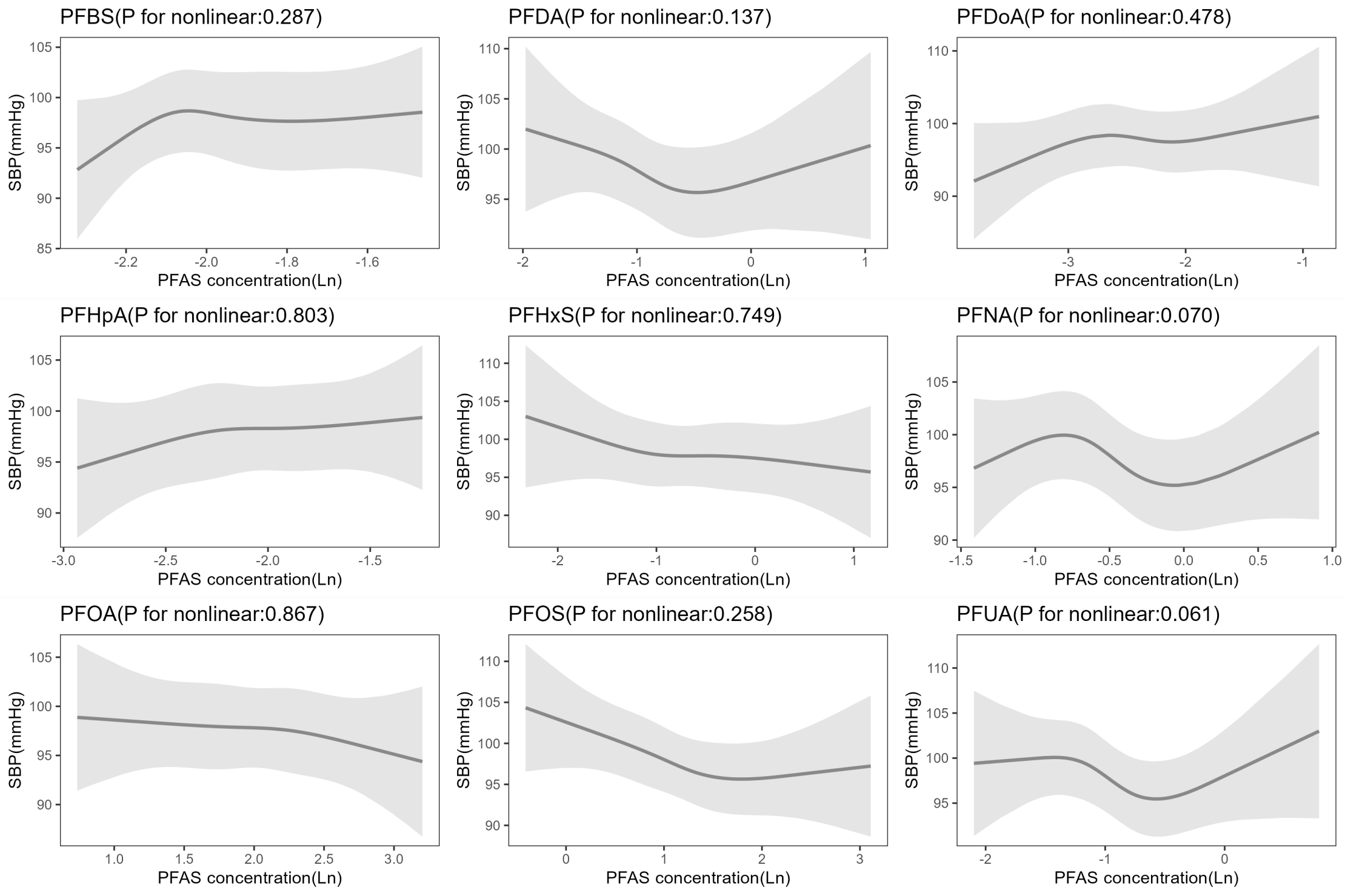


**Figure S5.** The restricted cubic spline of umbilical PFAS concentrations with children SBP.

The solid lines indicated the predicted SBP derived from restricted cubic spline regression model with 4 knots at the 5th, 35th, 65th and 95th percentiles of SBP. The shadow indicated the 95%CIs. Tests for non-linearity were conducted by using analysis of variance tests.

*indicates the non-linear P value<0.05.

Models were adjusted for household income, educational levels, GDM, HDP, drink history, passive smoke history, age of mom, birthweight, sex and BMI of children. BMI: body mass index, SBP: systolic blood pressure,HDP: hypertensive disorders in pregnancy, GDM: gestational diabetes mellitus.


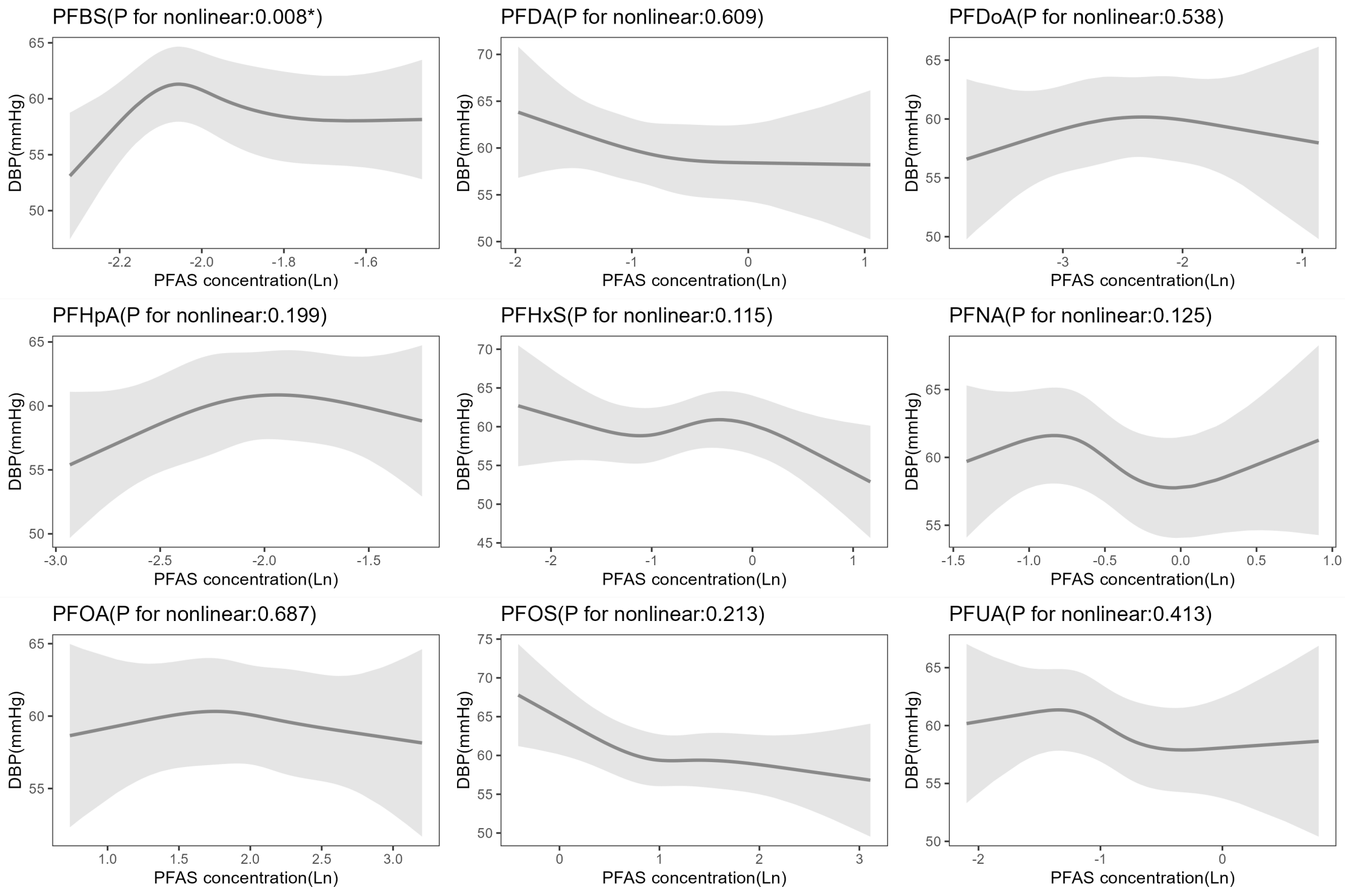


**Figure S6.** The restricted cubic spline of umbilical PFAS concentrations with children DBP.

The solid lines indicated the predicted DBP derived from restricted cubic spline regression model with 4 knots at the 5th, 35th, 65th and 95th percentiles of BP. The shadow indicated the 95%CIs. Tests for non-linearity were conducted by using analysis of variance tests.

*indicates the non-linear P value<0.05.

Models were adjusted for household income, educational levels, GDM, HDP, drink history, passive smoke history, age of mom, birthweight, sex and BMI of children. BMI: body mass index, DBP: diastolic blood pressure, HDP: hypertensive disorders in pregnancy, GDM: gestational diabetes mellitus.


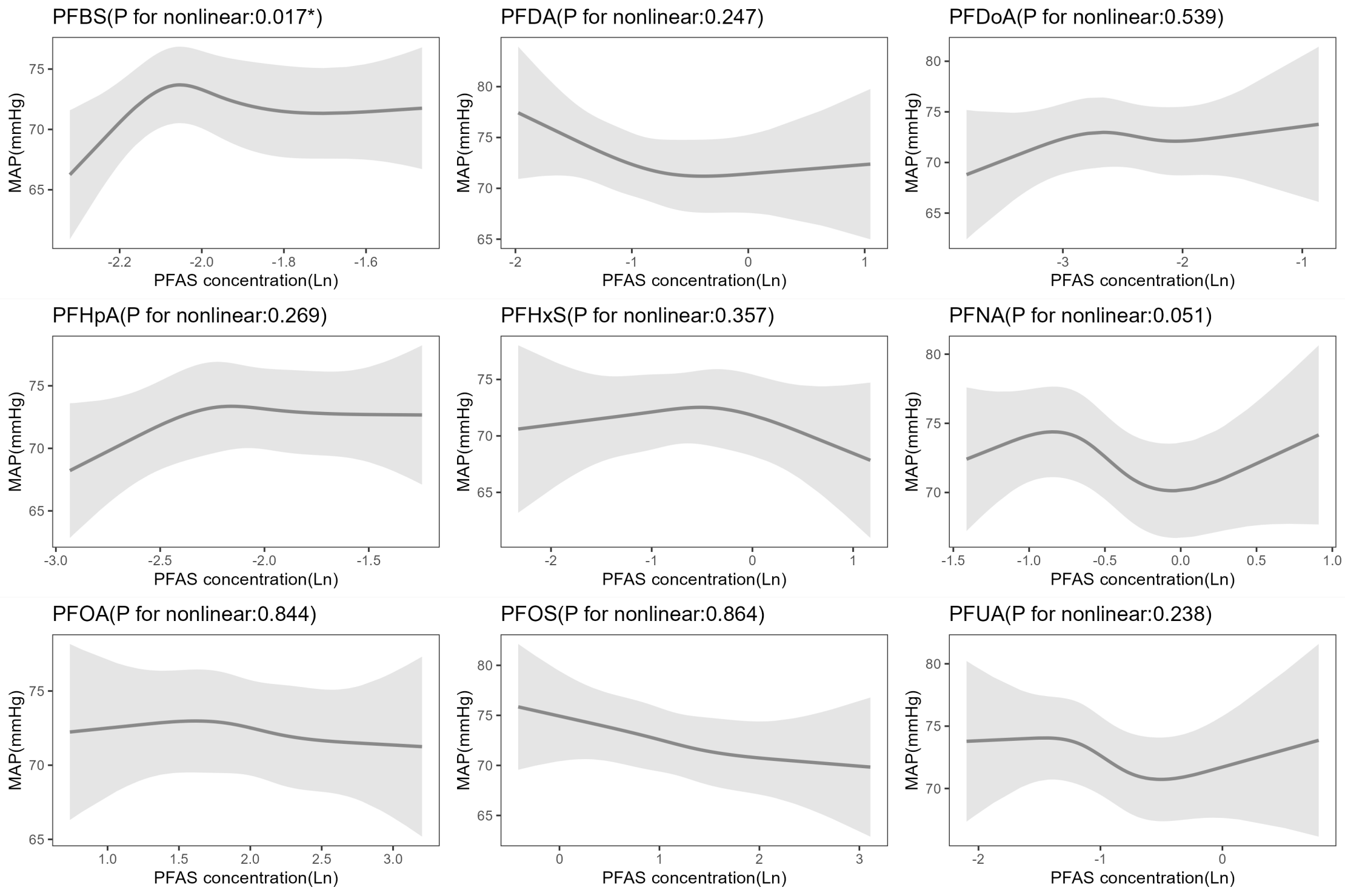


**Figure S7.** The restricted cubic spline of umbilical PFAS concentrations with children MAP.

The solid lines indicated the predicted MAP derived from restricted cubic spline regression model with 4 knots at the 5th, 35th, 65th and 95th percentiles of MAP. The shadow indicated the 95%CIs. Tests for non-linearity were conducted by using analysis of variance tests.

*indicates the non-linear P value<0.05.

Models were adjusted for household income, educational levels, GDM, HDP, drink history, passive smoke history, age of mom, birthweight, sex and BMI of children. BMI: body mass index, MAP: mean artery pressure, HDP: hypertensive disorders in pregnancy, GDM: gestational diabetes mellitus.


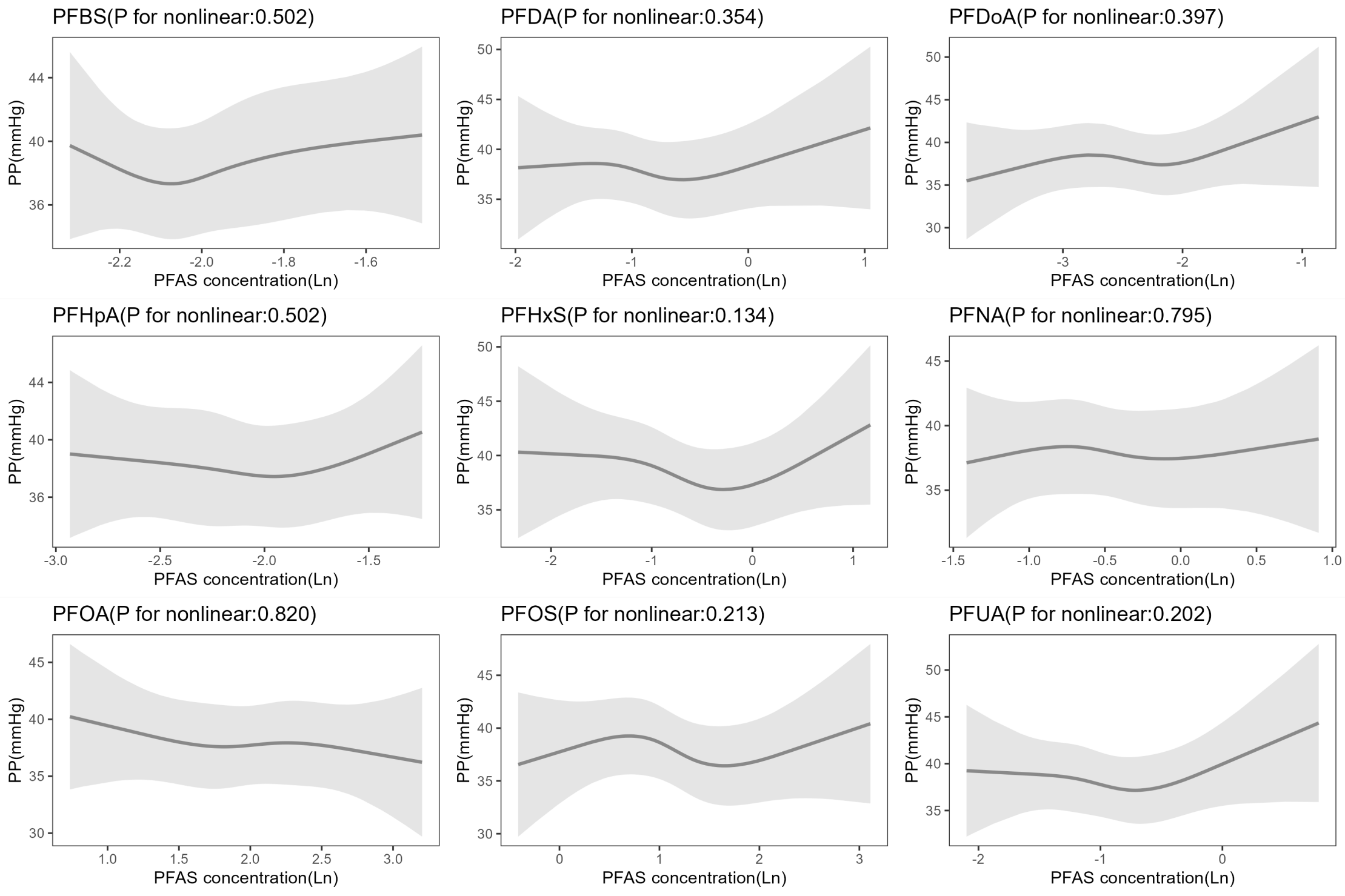


**Figure S8.** The restricted cubic spline of umbilical PFAS concentrations with children PP.

The solid lines indicated the predicted PP derived from restricted cubic spline regression model with 4 knots at the 5th, 35th, 65th and 95th percentiles of PP. The shadow indicated the 95%CIs. Tests for non-linearity were conducted by using analysis of variance tests.

*indicates the non-linear P value<0.05.

Models were adjusted for household income, educational levels, GDM, HDP, drink history, passive smoke history, age of mom, birthweight, sex and BMI of children. BMI: body mass index, SBP: systolic blood pressure, DBP: diastolic blood pressure, MAP: mean artery pressure, PP: pulse pressure, HDP: hypertensive disorders in pregnancy, GDM: gestational diabetes mellitus.

Table S3. Association of PFAS concentrations in umbilical cord blood plasma with children BP in different gender (male N=69, female N=60).

|  | SBP | | | | DBP | | | | MAP | | | PP | | | |  |
| --- | --- | --- | --- | --- | --- | --- | --- | --- | --- | --- | --- | --- | --- | --- | --- | --- |
|  | | Male | Female | P for interaction | Male | Female | P for interaction | Male | | Female | P for interaction | | Male | Female | P for interaction | |
| PFDA | | **-4.92(-8.81,-1.04)** | -0.59(-4.85,3.68) | 0.295 | -3.04(-6.44,0.36) | -3.08(-6.67,0.51) | 0.375 | **-3.73(-6.87,-0.62)** | | -2.14(-5.53,1.25) | 0.954 | | -1.88(-5.56,1.79) | 2.49(-0.80,5.78) | **0.039** | |
| PFDoA | | -0.82(-5.24,3.59) | **4.95(1.36,8.61)** | **0.008** | 1.59(-2.12,5.29) | 0.15(-3.24,3.54) | 0.257 | 0.26(-3.28,3.80) | | 1.36(-1.77,4.50) | 0.744 | | -2.41(-6.27,1.45) | **4.81(2.09,7.57)** | **0.000** | |
| PFHpA | | 1.14(-5.07,7.35) | **5.54(0.34,10.77)** | 0.095 | -1.21(-6.44,4.03) | **4.84(0.30,9.34)** | 0.133 | -0.03(-5.02,4.95) | | 3.94(-0.33,8.20) | 0.220 | | 2.35(-3.13,7.83) | 0.73(-3.57,5.04) | 0.665 | |
| PFHxS | | -0.70(-3.69,2.30) | -1.08(-4.13,1.97) | 0.515 | -0.73(-3.26,1.79) | -0.39(-3.05,2.26) | 0.940 | -0.58(-2.98,1.83) | | 0.44(-2.04,2.91) | 0.321 | | 0.03(-2.64,2.71) | -0.68(-3.09,1.73) | 0.500 | |
| PFNA | | -3.84(-8.01,0.32) | 0.34(-4.47,5.14) | 0.228 | -2.01(-5.62,1.60) | -2.76(-6.85,1.33) | 0.395 | -2.80(-6.15,0.56) | | -2.09(-5.93,1.74) | 0.911 | | -1.83(-5.65,1.98) | 3.09(-0.59,6.77) | **0.026** | |
| PFOA | | -2.80(-7.09,1.50) | 0.41(-3.90,4.72) | 0.298 | -2.84(-6.43,0.75) | 1.08(-2.65,4.80) | 0.359 | -2.25(-5.70,1.20) | | 0.23(-3.25,3.72) | 0.468 | | 0.05(-3.85,3.94) | -0.67(-4.06,2.73) | 0.771 | |
| PFOS | | **-4.24(-7.82,-0.74)** | -2.23(-5.54,1.07) | 0.722 | -2.89(-5.96,0.18) | **-2.96(-5.73,-0.15)** | 0.245 | **-3.45(-6.29,-0.65)** | | -1.57(-4.24,1.11) | 0.938 | | -1.39(-4.73,1.95) | 0.71(-1.94,3.35) | 0.128 | |
| PFUA | | **-6.12(-10.52,-1.67)** | 0.01(-4.24,4.26) | 0.104 | -3.47(-7.39,0.46) | -3.20(-6.76,0.37) | 0.235 | **-4.52(-8.13,-0.97)** | | -1.39(-4.79,2.02) | 0.745 | | -2.63(-6.84,1.58) | 3.20(-0.01,6.42) | **0.002** | |
| PFBS | | -2.69(-14.17,8.80) | 8.10(-3.29,19.48) | 0.331 | -4.03(-13.68,5.61) | 2.84(-7.21,12.89) | 0.508 | -0.68(-9.90,8.54) | | 1.96(-7.42,11.34) | 0.967 | | 1.35(-8.89,11.58) | 5.26(-3.78,14.30) | 0.639 | |

Multiple linear regression models were used and adjusted for maternal and offspring factors including household income, educational levels, GDM, HDP, drinking history, passive smoking history and age of mom, birthweight, sex and BMI of children.

PFOA: perfluorooctanate, PFOS: perfluorooctane sulfonate, PFNA: perfluorononanoic acid, PFDA: perfluorodecanoic acid, PFUA: perfluoroundecanoic acid, PFHxS: perfluorohexanesulfonate, PFHpA: perfluoroheptanoic acid, PFDoA: perfluorododecanoic acid, PFBS: perfluorobutane sulfonate; BMI: body mass index, SBP: systolic blood pressure, DBP: diastolic blood pressure, MAP: mean artery pressure, PP: pulse pressure, HDP: hypertensive disorders in pregnancy, GDM: gestational diabetes mellitus.
